# Multi-Scale Remote-Sensing Phenomics Integrated with Multi-Omics: Advances in Crop Drought–Heat Stress Tolerance Mechanisms and Perspectives for Climate-Smart Agriculture

**DOI:** 10.3390/plants14182829

**Published:** 2025-09-10

**Authors:** Xiongwei Liang, Shaopeng Yu, Yongfu Ju, Yingning Wang, Dawei Yin

**Affiliations:** 1 Cold Region Wetland Ecology and Environment Research Key Laboratory of Heilongjiang Province, Harbin University, Harbin 150086, China; liangxiongwei007@163.com (X.L.); shaopengyu1972@163.com (S.Y.); juyongfu@163.com (Y.J.); 14b927013@hit.edu.cn (Y.W.); 2 State Key Laboratory of Urban Water Resource and Environment, Harbin Institute of Technology, Harbin 150086, China; 3 College of Agricultural Science, Heilongjiang Bayi Agricultural University, Daqing 163319, China

**Keywords:** drought–heat stress, remote sensing phenomics, multi-omics integration, ABA signaling pathway, climate-smart agriculture

## Abstract

Climate change is intensifying the co-occurrence of drought and heat stresses, which substantially constrain global crop yields and threaten food security. Developing climate–resilient crop varieties requires a comprehensive understanding of the physiological and molecular mechanisms underlying combined drought–heat stress tolerance. This review systematically summarizes recent advances in integrating multi-scale remote-sensing phenomics with multi-omics approaches—genomics, transcriptomics, proteomics, and metabolomics—to elucidate stress response pathways and identify adaptive traits. High-throughput phenotyping platforms, including satellites, UAVs, and ground-based sensors, enable non-invasive assessment of key stress indicators such as canopy temperature, vegetation indices, and chlorophyll fluorescence. Concurrently, omics studies have revealed central regulatory networks, including the ABA–SnRK2 signaling cascade, HSF–HSP chaperone systems, and ROS-scavenging pathways. Emerging frameworks integrating genotype × environment × phenotype (G × E × P) interactions, powered by machine learning and deep learning algorithms, are facilitating the discovery of functional genes and predictive phenotypes. This “pixels-to-proteins” paradigm bridges field-scale phenotypes with molecular responses, offering actionable insights for breeding, precision management, and the development of digital twin systems for climate-smart agriculture. We highlight current challenges, including data standardization and cross-platform integration, and propose future research directions to accelerate the deployment of resilient crop varieties.

## 1. Introduction

Climate change is exacerbating abiotic stresses such as drought and heat, which increasingly occur in combination and severely threaten global food security [1]. Drought and heat stress lead to extensive yield losses in major crops by disrupting plant physiological processes—drought limits water availability for photosynthesis, while heat waves damage enzymes and cellular structures [2]. For instance, elevated temperatures above optimal levels can suppress photosynthesis, induce excessive evapotranspiration, and cause oxidative damage, together reducing crop productivity [3,4]. Severe drought remains a major challenge for cereal and oilseed production. In critical crops like cereals and oilseeds, drought events have been shown to lead to significant yield reductions, as highlighted in assessments by the FAO and IPCC [1,5], it is consistently identified as one of the most damaging climate extremes for agriculture, with substantial yield reductions reported across diverse crops and regions, depending on crop type, geography, and stress intensity [6]. Each additional degree of warming is projected to increase the likelihood and severity of such compound drought–heat events, heightening the risk that photosynthetic gains cannot compensate for hydraulic and thermal limitations. Developing climate-resilient varieties capable of withstanding drought and heat stress is therefore a critical priority for safeguarding future global food and oilseed production.

Global economic costs of stress-related yield losses. Multi-country datasets show drought disasters reduce national cereal output by ~9–10% on average, with heat waves adding further losses and compounding impacts in low-latitude breadbaskets [7]. Meta-analyses estimate per-degree-warming yield penalties of ~−6% (wheat), −7% (maize), and ~−3% (rice/soybean) absent adaptation [7,8]. Accounting for price responses and supply chains, FAO assessments attribute tens of billions of USD in agricultural losses annually to weather extremes, with compound drought–heat events increasingly dominant [1,5].

Abiotic stress encompasses diverse non-living constraints, with drought and heat being the most critical for global agriculture. Drought reduces stomatal conductance and leaf water potential, triggering osmotic adjustment (e.g., proline, soluble sugars) and hydraulic remodeling (root allocation, aquaporins) [9,10]. Heat adds thermal load that can overwhelm evaporative cooling, elevating canopy and leaf temperatures and promoting reactive oxygen species (ROS) accumulation [11,12]. Under combined drought–heat conditions, plants must balance carbon gain against hydraulic safety and thermal homeostasis. Tolerance emerges when adaptive responses stabilize key functional traits—such as canopy temperature depression (CTD), chlorophyll fluorescence (Fv/Fm), stem water potential, and membrane integrity—that are quantifiable by remote sensing (thermal, red-edge/PRI, solar-induced fluorescence, SIF) and linkable to omics signatures (e.g., ABA–SnRK2 signaling, HSF–HSP chaperones, antioxidant pathways) [13,14]. Notably, co-stress responses are often emergent and non-additive—due to hormone crosstalk, amplified ROS bursts when evaporative cooling is constrained, elevated proteostasis demands (HSF–HSP), and hydraulic–thermal feedbacks—producing greater injury than either stress alone and justifying explicit phenotyping under synchronized water and thermal deficits [2,13,14,15]. This integrated view yields measurable indicators for selection and management under field variability [16].

At the molecular level, several core signaling pathways mediate plant responses to drought and heat. The abscisic acid (ABA) signaling pathway is central in drought tolerance: under water deficit, ABA accumulates and initiates a signaling cascade via *pyrabactin resistance1/PYR-like* (PYR/PYL) receptors, protein phosphatase 2C (*PP2C*) inactivation, and SnRK2 kinase activation, which leads to expression of numerous drought-responsive genes and stomatal closure [17,18]. Small-molecule studies have confirmed the pivotal role of ABA pathways; for example, synthetic ABA receptor agonists that mimic ABA can significantly improve drought resilience in crops by activating downstream stress responses. In parallel, the heat shock factor–heat shock protein (HSF–HSP) network is crucial for heat tolerance. Upon high temperatures, heat shock transcription factors (e.g., *HsfA1, HsfA2*) are released from chaperones and upregulate heat shock proteins (HSP70, HSP90, HSP101, etc.), which function as molecular chaperones to prevent protein unfolding and aggregation [19,20]. This HSF–HSP system equips cells with thermotolerance by stabilizing proteins and refolding denatured enzymes during heat stress. Notably, drought and heat signaling pathways cross-talk: ABA-responsive element binding factors can also regulate genes involved in heat resistance [21], and heat stress can elevate ABA levels that in turn modulate stress-responsive genes [22]. Both stresses also converge on ROS signaling—drought and heat induce accumulation of ROS like hydrogen peroxide, which at moderate levels serve as secondary messengers to trigger defense genes, but at high levels cause oxidative damage if not scavenged by antioxidant enzymes [23,24]. Therefore, an interplay between hormonal signals (ABA and others), chaperone systems (HSPs), and redox homeostasis underpins the physiological and molecular mechanisms of drought–heat tolerance.

**Figure 1:**
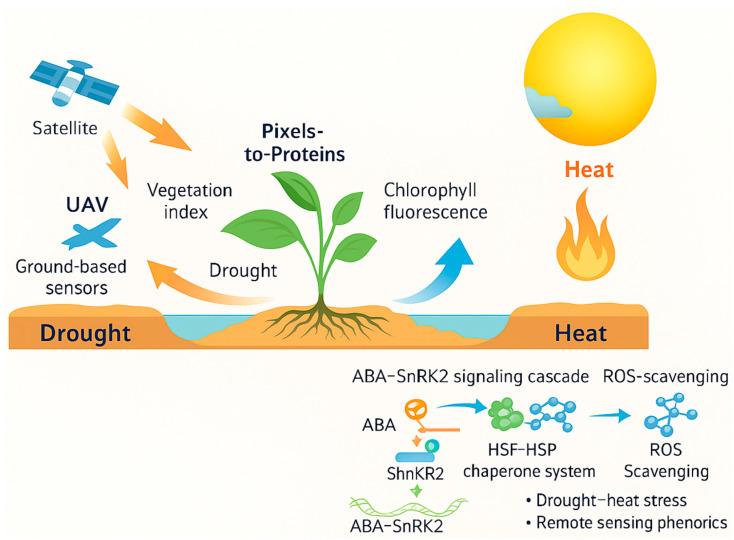
Integrating Remote Sensing and Multi-Omics to Dissect Crop Tolerance to Combined Drought and Heat.

To build climate-resilient crop systems, plant scientists are exploiting new technologies to better understand and harness these complex tolerance mechanisms. Traditional breeding for drought or heat tolerance has been challenging due to the multigenic nature of these traits and difficulty of phenotyping under field stress conditions. However, recent advances in remote sensing-based high-throughput phenotyping (phenomics) and multi-omics profiling (genomics, transcriptomics, proteomics, metabolomics) are revolutionizing how we study plant stress responses [25]. Phenomics enables the rapid measurement of plant traits (morphology, physiology, performance) at scale using sensors and imaging, providing rich datasets on how different genotypes perform under stress in realistic environments. Meanwhile, omics technologies allow dissection of the genetic and biochemical underpinnings of stress tolerance by surveying the genome and downstream molecules (RNA, proteins, metabolites) comprehensively. Integrating phenomics and omics information—effectively connecting observable traits to specific genes, proteins, and metabolites—offers a powerful approach to identify stress-adaptive mechanisms and candidate tolerance genes. For example, high-throughput field imaging can pinpoint genotypes with cooler canopies or sustained greenness under drought, and genome-wide association studies (GWAS) can then map genomic loci linked to those phenotypic traits [26]. Likewise, transcriptomic and proteomic analyses of stress-exposed plants reveal the activation of protective pathways (like the ABA–SnRK2 or HSF–HSP modules), which can be correlated with observed stress tolerance phenotypes. By connecting these scales—from satellite pixels capturing field performance down to proteins and genes governing stress responses—researchers are developing holistic strategies to breed and manage crops for improved drought–heat resilience. This review provides an overview of these advances in multi-scale phenomics and multi-omics integration and discusses how they are being translated into climate-smart agriculture practices (Figure 1). Figure 1 operationalizes the “pixels-to-proteins” pipeline: spectral/thermal signals map to field-level traits (e.g., CTD, PRI, SIF), align with tolerance phenotypes, and connect—via GWAS/TWAS and targeted metabolomics—to causal pathways (ABA/HSF–HSP/ROS), closing the loop to breeding priorities and decision thresholds. This schematically illustrates how remote sensing and omics layers converge from canopy to molecular scale, emphasizing the integrative nature of this review.

## 2. Multi-Scale Remote-Sensing Phenomics

**Figure 2:**
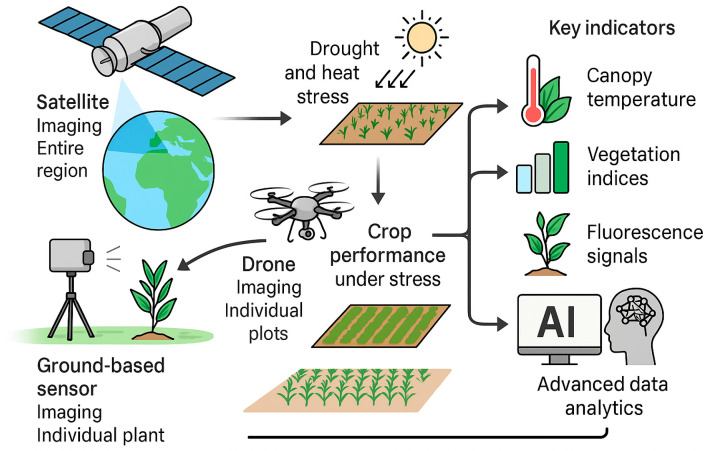
Satellite-to-Ground Phenotyping Workflow for Heat and Drought Monitoring.

High-throughput plant phenomics leverages remote sensing technologies across multiple scales—from satellites imaging entire regions to drones and ground-based sensors imaging individual plots or plants—to capture crop performance under stress in a rapid, quantitative manner. In the context of drought and heat stress tolerance, multi-scale remote-sensing phenomics provides invaluable traits such as canopy temperature, vegetation indices, and fluorescence signals that serve as proxies for plant health and stress levels [27,28]. This section details the major phenotyping platforms and sensors, the key indicators they measure, and the role of advanced data analytics (AI and machine learning) in extracting stress-relevant information. Schematic of a phenotyping pipeline that integrates satellite, drone, and ground-based imaging to quantify crop performance under drought and heat. Key indicators—canopy temperature, vegetation indices, and fluorescence signals—are extracted and analyzed with AI/ML to derive stress-relevant traits (Figure 2).

### 2.1. Phenotyping Platforms: Satellite, Airborne, UAV, and Ground-Based

Satellite remote sensing offers a macro-scale perspective on crop performance, proving essential for regional drought monitoring and yield forecasting. Operational sensors such as MODIS, Landsat, and Sentinel-2 capture vegetation health through multispectral imagery and thermal infrared measurements. For example, satellite-derived drought indices have monitored severity and crop impacts over multi-decade periods [29,30]. A study from 2001 to 2020 utilized MODIS data to map drought stress in Sichuan Province croplands via vegetation indices, revealing spatiotemporal patterns of impacts [31]. Satellites’ key advantage is comprehensive coverage, regularly scanning vast agricultural regions to detect stress hotspots in real fields and bridge-controlled experiments with farm-scale outcomes. Limitations include moderate spatial resolution (10–30 m for Sentinel/Landsat, coarser for MODIS) and reliance on clear skies, as clouds obstruct optical sensors. Nevertheless, satellites are increasingly paired with field trials to assess genotype performance under authentic stress, with emerging radar technologies (e.g., Sentinel-1) addressing cloud issues. Typical GSD is 10–30 m (S2/L8) with 5–16 d revisit; dominant failure modes include cloud contamination and mixed pixels. BRDF correction and cross-dates normalization are recommended for time-series analyses.

Airborne platforms, including manned aircraft or helicopters with sensors, provide an intermediate scale with sub-meter resolution over trial fields, covering areas beyond ground systems’ reach. They have aided breeding programs by phenotyping drought responses in large plots of wheat or beans [32]. One investigation deployed both unmanned aerial systems and high-altitude aircraft to acquire high-resolution multispectral imagery for dry bean phenotyping, correlating canopy cover and temperature with drought tolerance rankings [33]. Airborne methods support custom sensor configurations, such as hyperspectral imagers for detailed stress detection, and have been valuable for capturing data in research stations, especially pre-drone era. However, they are logistically demanding, expensive, and less flexible in timing compared to UAVs, requiring specialized operations.

Unmanned aerial vehicles (UAVs), or drones, have become central to field phenomics due to their high flexibility, fine resolution (cm-level), and relative affordability. UAVs accommodate multispectral cameras, thermal sensors, LiDAR, and hyperspectral imagers, allowing on-demand flights to target growth stages or stress onsets. In drought and heat studies, they measure NDVI, canopy temperature, plant height, and biomass across numerous plots per flight [8,34]. For wheat under drought, UAV NDVI and temperature imagery identified stay-green phenotypes and cooler canopies linked to better yields [35,36]. Studies show UAVs achieve similar or superior precision to manual measurements for NDVI, capturing whole-plot variation [37]. Under heat, thermal UAVs pinpoint efficient transpirational cooling, suggesting deeper roots [38]. Applications extend to rice, where drone RGB tracked senescence dynamics tied to genetics [39], and sorghum, where biomass data informed GWAS for resilience loci [40]. UAVs democratize phenomics, enabling small programs to collect high-throughput data, though regulatory and data processing challenges persist.

Ground-based phenotyping systems, ranging from handheld devices and portable carts to fixed installations, deliver the highest precision and support sensors unsuitable for aerial use, like chlorophyll fluorometers or gas-exchange analyzers. Phenomobiles (sensor-mounted vehicles) scan plots for reflectance, fluorescence, or architecture; one equipped with multispectral and thermal sensors identified drought-tolerant wheat via cooler temperatures [41]. In controlled environments, automated imaging captures wilting kinetics or growth under imposed stresses [42]. A low-cost greenhouse system with ML analyzed drought tolerance in grasses [43]. Ground measurements validate remote indices, e.g., correlating NDVI to leaf water potential or photosynthesis [44]. Despite lower throughput and labor needs, they complement by providing calibration for multi-scale integration.

**Figure 3:**
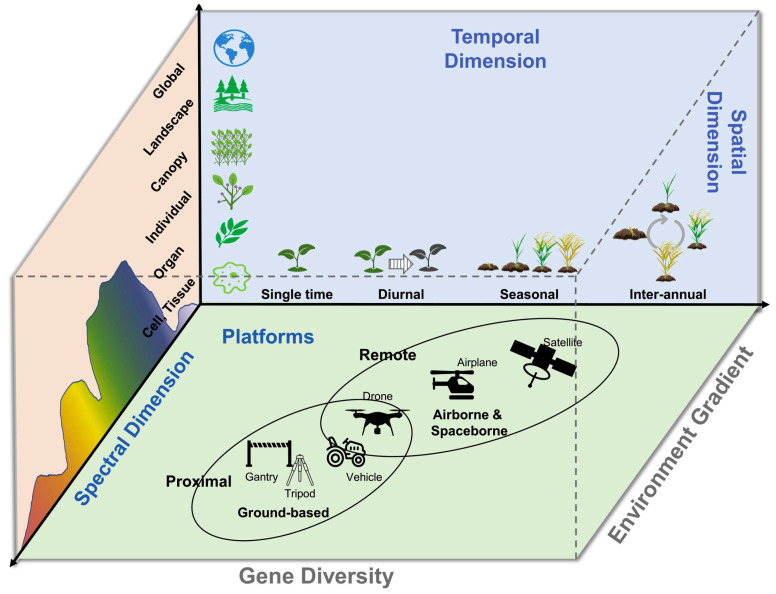
Schematic overview of proximal and remote sensing platforms in plant phenomics, including satellite, airborne, UAV, and ground-based systems [45]. Copyright 2022 Elsevier.

Collectively, these platforms synergize satellites for breadth, airborne/UAVs for detail, ground for validation, fostering holistic crop assessments under drought and heat. As illustrated in Figure 1, multi-scale phenotyping platforms enable complementary data acquisition for comprehensive crop stress assessment (Figure 3) [45]. Practical examples highlight the power of these platforms. For instance, MODIS time-series vegetation indices were applied to monitor stay-green expression on wheat, revealing delayed senescence phenotypes that aligned with yield stability under terminal drought [46,47]. Such coarse-resolution monitoring provides a regional context and identifies hotspots that can later be examined with UAV flights. UAV thermal and hyperspectral imaging then resolve canopy-level responses—such as midday canopy cooling (CTD) and pigment dynamics (PRI/red-edge)—at the plot scale [48], while ground-based measurements provide calibration/validation (Cal/Val) through direct assessments of leaf water potential, stomatal conductance, or chlorophyll fluorescence (Fv/Fm) [49]. Together, these platforms enable a multi-scale pipeline: satellites for breadth and trend detection, UAVs for high-resolution trait dissection, and ground systems for mechanistic fidelity, bridging discovery from landscape variability to genotype-level selection.

### 2.2. Key Phenotypic Indicators of Drought and Heat Stress

Remote-sensing phenomics relies on indicators that signal plant physiological status under stress, enabling early detection and genotypic screening. These metrics—spectral, thermal, and structural—proxy traits like water-use efficiency and photosynthesis. Multi-indicator integration enhances accuracy, as no single metric captures complexity. Below, we outline key indicators, their basis, applications, and genetics.

Normalized Difference Vegetation Index (NDVI), (NIR − Red)/(NIR + Red), correlates with biomass and chlorophyll. Under drought/heat, NDVI drops from senescence; “stay-green” genotypes sustain it, linking to yields in cereals [50,51]. NDVI proxies adaptations like rooting; QTLs control it in wheat/maize under drought [52]. Derivable across platforms, it is foundational but saturates in dense canopies.

Canopy temperature (CT), via thermal sensors, reflects transpirational cooling. Drought closes stomata, raising CT; cooler canopies indicate tolerance [53]. UAV mapping in wheat ties lower CT to roots/yields [54]. Canopy temperature depression (CTD) normalizes; CIMMYT selects via infrared for stressed environments [55]. Sensitive to microclimate, CT benefits contextual fusion.

Chlorophyll fluorescence (Fv/Fm = (Fm − F0)/Fm) assesses PSII yield, declining from photoinhibition [56]. Fluorometry identifies heat-tolerant wheat sustaining Fv/Fm [57]. Solar-induced fluorescence (SIF) proxies canopy photosynthesis, dropping in drought.

Advanced indices target specifics: Photochemical Reflectance Index (PRI = (R531 − R570)/(R531 + R570)) detects xanthophyll shifts for efficiency; NDWI estimates hydration. Red-edge senses chlorophyll early. Hyperspectral in stressed wheat outperformed NDVI for yields [58]. Table 1 lists some commonly used indices (e.g., NDVI, PRI, Normalized Difference Water Index) along with their formulae and what aspect of stress they reflect. By using hyperspectral sensors (which capture continuous spectra), researchers can calculate dozens of indices or even apply machine learning to find the most predictive wavelength features for stress tolerance [59]. For instance, in maize, machine learning on hyperspectral data identified specific spectral bands linked to drought susceptibility, which aligned with known pigment and water absorption features. Table 1 provides an overview of common platforms and sensor types, their typical spatial/temporal resolution, and example stress indicators obtainable from each. Table 1 contrasts major platforms (satellite, UAV, ground) by resolution/revisit, strengths, limitations, and Cal/Val essentials; Table 2 lists representative indicators and what aspect of stress they reflect.

**Table 1:** Comparative overview of phenotyping platforms for drought–heat detection.

Platform	Resolution	Key Indicators (Examples)	Strengths	Limitations	Representative Applications in Drought–Heat Detection
Satellite (e.g., Sentinel-2, MODIS, Landsat)	Low (10–500 m); revisit 5–16 d	NDVI, LST, NDWI	Regional coverage; multi-decadal time series; free/low cost	Cloud dependence; coarse resolution; limited genotype inference	Sentinel-2 NDVI & LST mapped regional drought severity and yield decline in wheat [60]; MODIS time-series revealed stay-green phenotypes in Sichuan wheat [31]
UAV/Drones	High (cm-level, on-demand)	CTD, PRI, hyperspectral reflectance, RGB/LiDAR	Plot-level precision; captures transient stress	Flight regulations; short battery life; heavy data pipelines	UAV NDVI + canopy temperature identified drought-tolerant wheat genotypes with cooler canopies [61]; UAV hyperspectral (red-edge, PRI) detected pigment dynamics under heat stress in maize [62]
Ground-based sensors (tripods, gantries, handhelds)	Very high (mm–cm)	Fv/Fm, PRI, leaf water potential	Highest mechanistic fidelity; essential for Cal/Val	Labor-intensive; very limited area	Porometry + leaf water potential validated UAV CTD signals in wheat [63]; chlorophyll fluorescence (Fv/Fm) distinguished heat-tolerant rice cultivars [49]
Proximal phenotyping vehicles (e.g., carts, tractor-based)	Medium (m-level)	Multispectral/LiDAR imaging	Higher throughput than handheld; bridges plots to fields	Logistic constraints; less scalable than UAVs or satellites	Phenomobile multispectral imaging quantified soybean canopy traits under drought, supporting QTL mapping [64]

Table 1 highlights the coverage–precision–scalability trade-off across platforms. Satellites provide unmatched coverage for early warning but lack genotype-level detail. UAVs capture fine-scale, transient stress signals yet face regulatory and processing limits. Ground systems offer mechanistic fidelity and serve as the Cal/Val standard but cover small areas, while proximal vehicles bridge UAV and ground scales. In practice, integrating satellites for breadth, UAVs for plot-level traits, and ground/proximal sensors for validation provides a robust pipeline for drought–heat phenotyping.

**Figure 4:**
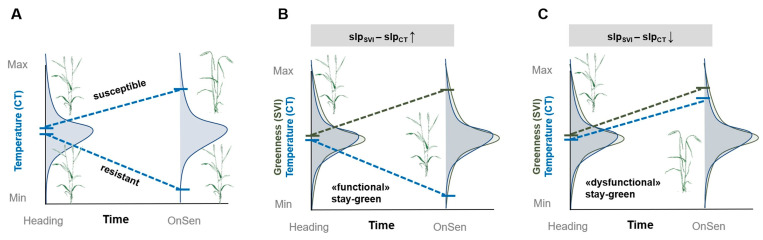
Contrasting stay-green strategies under drought–heat stress: effects on canopy temperature, photosynthesis, and chlorophyll dynamics [68]. Copyright 2021 Elsevier.

Where appropriate, satellite-derived indices can be downscaled and calibrated to plot scale using UAV subsets, enabling trial-wide monitoring when flight capacity is limited. For biomass and senescence traits, UAV RGB/LiDAR quantifies fractional cover and canopy height, and stress accelerates senescence. In wheat, image segmentation produced indices associated with heat QTLs [59], while rice time-series dynamics revealed drought-related genetic effects [65]. Combining spectral greenness with canopy temperature—e.g., NDVI + canopy temperature depression (CTD)—improves screening accuracy [66,67]. As illustrated in Figure 4, joint temporal trajectories of canopy temperature and greenness distinguish drought-avoidance and stay-green strategies in wheat [68].

Figure 4 illustrates contrasting physiological strategies influencing drought–heat resilience. Panel A shows that tolerant genotypes often maintain lower canopy temperatures (more negative canopy temperature depression, CTD) due to sustained transpiration, whereas susceptible lines exhibit warmer canopies and accelerated stress onset. Panel B depicts differences in photosynthetic efficiency: stay-green lines sustain higher rates over time, while sensitive lines experience sharp declines during heading and grain filling. Panel C highlights chlorophyll dynamics, where delayed senescence (“stay-green”) preserves green leaf area and supports assimilate supply under stress, in contrast to rapid yellowing in susceptible lines. Collectively, Figure 4 underscores how canopy cooling, photosynthetic stability, and chlorophyll retention serve as complementary mechanisms underpinning stress tolerance, and how these can be captured through integrated remote-sensing phenomics. This figure emphasizes that no single trait ensures resilience: canopy cooling, photosynthetic stability, and delayed senescence interact to determine yield outcomes. Importantly, each trait also has trade-offs—prolonged stay-green may delay remobilization under mild stress, while high transpirational cooling can increase water demand.

### 2.3. AI and Machine Learning for Trait Extraction and Sensor Integration

The surge in high-throughput phenomics produces expansive datasets, encompassing hyperspectral imagery, temporal sequences, and outputs from diverse sensors, demanding advanced tools for insightful analysis [69,70]. Artificial intelligence (AI) and machine learning (ML) have become essential in deriving valuable insights from these intricate datasets, enabling the identification of stress-associated traits in crops facing drought and heat challenges. Spanning supervised and unsupervised frameworks, these approaches range from traditional regression techniques to cutting-edge deep learning models, effectively tackling core phenotyping hurdles.

For trait extraction and image processing, convolutional neural networks (CNNs) stand out in handling visual inputs to measure morphological and physiological attributes. CNNs proficiently segment plant structures, identify senescence indicators, or categorize stress manifestations with superior accuracy. One exemplary case utilized a deep learning framework on UAV-captured maize leaf images to enumerate microscopic bulliform cells, facilitating large-scale evaluation of drought-adaptive features that were formerly confined to labor-intensive microscopy [71]. Likewise, ML-based segmentation in heat-stressed wheat quantified foliar senescence progression, linking these indices to yield resilience and aiding genomic breeding strategies [72].

In predictive analytics, ML harnesses sensor features to anticipate stress effects and yield projections. Methods like random forests and partial least squares regression dissect hyperspectral profiles to infer biochemical markers, such as chlorophyll and hydration levels, serving as surrogates for stress dynamics [73]. Ensemble frameworks in wheat under drought, amalgamating various spectral metrics, forecasted grain output with high fidelity, offering noninvasive aids for preliminary selection in breeding programs [74]. Moreover, classification models delineate stress categories—e.g., differentiating drought from nutritional deficits—via integrated thermal and spectral data [75].

ML enhances sensor fusion by merging disparate data modalities, boosting trait precision. Multivariate algorithms blend hyperspectral reflectance (reflecting canopy vitality) with thermal scans (depicting evaporative cooling) to estimate traits like stomatal conductance in drought-affected wheat [76,77]. Such integration delineates comprehensive stress profiles, surpassing isolated sensor utilities. Temporal scrutiny, via recurrent neural networks, illuminates dynamic patterns, including NDVI degradation post-drought initiation, unveiling attributes like recuperative capacity.

Beyond data handling, ML reveals emergent markers; for instance, importance assessments in soybean hyperspectral analyses pinpointed mid-infrared wavelengths tied to foliar hydration, refining stress metrics [74]. Holistic workflows, where AI-derived traits inform genome-wide association studies (GWAS) in sorghum, expedite resilience gene identification [78].

Ultimately, AI and ML convert unprocessed phenomics inputs into biologically meaningful traits, amplifying stress phenotyping for breeding resilient varieties. Progress in interpretable AI will better synchronize models with underlying biology, mitigating opacity issues and broadening utility in variable field settings.

### 2.4. Sensor Integration and High-Throughput Stress Phenotyping

An important trend in field phenotyping for climate resilience is the integration of multiple sensor modalities to capture the different dimensions of plant stress response. No single measurement can fully characterize a complex trait like drought tolerance; thus, researchers increasingly combine, for example, spectral and thermal imaging, or imaging and soil sensors, to obtain a holistic view. A prime illustration is the integration of hyperspectral and thermal UAV imagery in wheat: by simultaneously measuring canopy reflectance (which can indicate chlorophyll content, nitrogen status, etc.) and canopy temperature (indicating water status), one study was able to identify genotypes that maintained both high greenness and low temperature under drought—a desirable combination [79]. This dual-sensor approach led to the discovery of genomic loci associated with those combined traits, underscoring how sensor integration can sharpen the phenotype definition for genetic analysis [80].

Another example is combining fluorescence with imaging. New field phenotyping rigs can measure pulse-modulated chlorophyll fluorescence alongside spectral reflectance. In a drought experiment on maize, simultaneous recording of fluorescence (Fv/Fm) and NDVI provided insight that some genotypes had high NDVI (stayed green) but low Fv/Fm (photoinhibition) under stress, whereas a few maintained both—the latter were truly tolerant [81]. Without measuring fluorescence, one might wrongly assume all green genotypes are fine; the integrated sensing revealed subtler differences in photosynthetic function.

Beyond plant-mounted sensors, integration often includes environmental sensors (weather stations, soil moisture probes) to precisely characterize the stress each plot experiences. This contextual data is crucial: for instance, linking a plot’s canopy temperature to its soil moisture and atmospheric vapor pressure deficit helps differentiate whether a high temperature is due to genotype or simply due to variation in micro-environment. In phenomics experiments, networks of soil moisture sensors or IoT-based systems (like the iPOT system for automated drought imposition [82]) ensure each genotype’s stress level is known, allowing more accurate association of phenotypes with genetic differences.

The concept of sensor fusion extends to merging data across scales. A notable approach is calibrating coarser-resolution satellite indices using finer drone data. For instance, if a breeding program can only obtain satellite NDVI but at 10 m resolution (covering many plots), they might use a drone on a subset of fields to develop a calibration model translating satellite signals into plot-level values or stress ratings. This multi-scale synergy can enable larger-scale monitoring of trials when drone coverage is limited.

Integration also implies combining aboveground and belowground phenotyping. While remote sensing focuses on aboveground traits (canopy, leaves), root traits are pivotal in drought tolerance. Some studies have integrated shovelomics or root imaging (e.g., via soil coring or ground-penetrating radar) with aboveground phenotypes. One study mapped root architectural traits in wheat and compared them with UAV-based drought indices, finding that deeper roots were associated with cooler canopies and higher yield in drought, tying the belowground mechanism to the aboveground signature [83,84]. Although root phenotyping remains challenging, these integrated efforts highlight how multi-faceted true drought tolerance is.

In summary, multi-scale remote-sensing phenomics is most powerful when multiple complementary sensors are used and their data combined. By capturing various physiological signals (greenness, temperature, fluorescence, structure) and integrating them with environmental measurements, researchers can obtain a comprehensive stress phenotype for each genotype. This improves the identification of truly resilient genotypes and the genetic loci underlying complex traits, as demonstrated in numerous high-throughput studies across crops [85,86]. The next sections will transition from phenotypes to the multi-omics underpinnings of drought–heat tolerance and then discuss how we integrate across genotype (G), environment (E), and phenotype (P) in emerging “pixels-to-proteins” frameworks.

Table 2 compares widely used indicators—NDVI, canopy temperature depression (CTD), Fv/Fm, plant height, and stay-green index—covering their physiological basis, measurement modality, and typical correlations with yield under combined drought–heat stress. Across crops, NDVI and CTD show consistently strong associations with yield maintenance, making them robust, scalable proxies for field screening, whereas Fv/Fm provides an early mechanistic signal at smaller plot scales. These patterns, drawn from multiple studies, offer practical guidance for trait prioritization.

Case study—compound drought–heat detection using the platforms in Table 1. Design: split-plot (well-watered vs. deficit) × passive heating (+2–4 °C at midday), n ≈ 24 plots (≥4 genotypes). Sensing: UAV thermal to compute canopy temperature depression (CTD = T_air_ − T_canopy_) and RGB/hyperspectral for structure/greenness at solar noon and late afternoon on stress days; co-sample leaf water potential and stomatal conductance within ±2 h. Thresholds: flag compound stress when CTD falls to ≤+1 °C at noon and the stay-green/greenness signal declines on ≥2 flights; confirm with Ψ_leaf_ < −1.5 MPa. Outputs: (i) a selection index combining CTD with greenness dynamics; (ii) prescriptions (pre-irrigation or temporary shading at booting–flowering); (iii) validation with yield components and membrane stability. (Thresholds require site-year calibration.) Threshold and flight-time choices follow recent UAV thermal drought-monitoring practice and calibrated ΔT/CWSI ranges in winter wheat; midday/early-afternoon flights maximize discriminatory power [46,87].

**Table 2:** Phenotypic indicators of drought–heat tolerance: physiology, measurement, correlation with yield (R), and representative applications. CTD = T_air_ − T_canopy_ (larger CTD = cooler canopy).

Indicator	Physiological Basis	Measurement Type	Correlation with Yield Under Stress	Crops Reported	Representative Findings
NDVI (Normalized Difference Vegetation Index)	Canopy greenness/biomass	Multispectral reflectance	High (R = 0.6–0.8)	Wheat, Maize, Rice	NDVI decline correlated with yield loss in maize under South Asian drought trials [88]
CTD (Canopy Temperature Depression = T_air_ − T_canopy_)	Transpirational cooling	Thermal imaging	High (R = 0.6–0.8)	Wheat, Sorghum	Cooler canopies identified tolerant wheat lines across multi-location trials [68]
Fv/Fm (maximum quantum efficiency of PSII)	Photosystem II efficiency	Chlorophyll fluorescence sensor	Moderate (R = 0.4–0.6)	Rice, Soybean	Heat-tolerant genotypes maintain higher Fv/Fm under stress [89]
Plant height	Growth/vigor proxy	RGB or LiDAR	Variable (context-dependent)	Maize, Sorghum	Height alone inconsistent; combined with NDVI improves prediction [90]
Stay-green index	Delayed senescence	NDVI time-series	Moderate to High (R = 0.5–0.7)	Wheat, Pearl Millet	Stay-green genotypes showed improved grain filling under heat stress [89]

Note: R ranges are indicative and context dependent. We use CTD = T_air_ − T_canopy_ (larger CTD = cooler canopy → typically positive correlation with yield). Thermal flights should be made near solar noon; NDVI can saturate at high LAI; thresholds require site-year calibration.

## 3. Multi-Omics Dissection of Drought–Heat Tolerance

**Figure 5:**
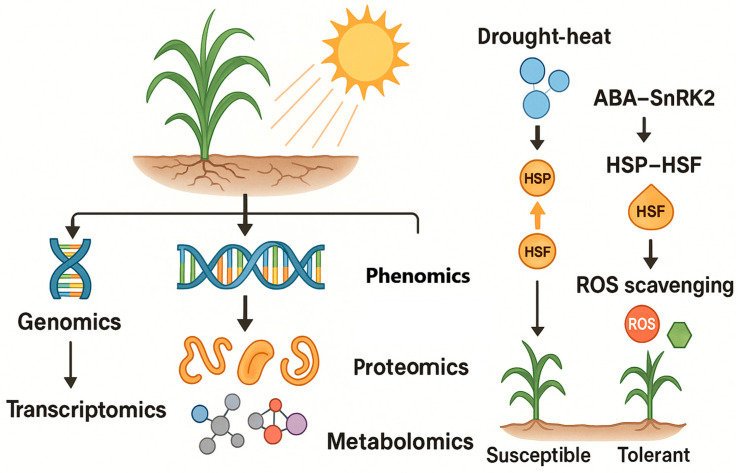
Multi-Omics Framework for Drought–Heat Tolerance: From Genomes to Metabolites and Key Protective Pathways.

Understanding and improving crop tolerance to drought and heat requires delving into the biological mechanisms at the genomic, transcriptomic, proteomic, and metabolomic levels. High-throughput “omics” approaches enable comprehensive dissection of these mechanisms by identifying the genes, gene expression changes, proteins, and metabolites that differentiate stress-tolerant vs. susceptible genotypes. In recent years, multi-omics studies have greatly advanced our knowledge of drought–heat stress responses, often revealing complex networks of signaling pathways and protective molecules involved in tolerance [91,92]. This section summarizes key contributions of each omics level—genomics, transcriptomics, proteomics, and metabolomics—to unraveling drought/heat tolerance, with emphasis on some well-known pathways like ABA–SnRK2, HSP–HSF, and ROS scavenging, and highlights how these layers interact (Figure 5).

### 3.1. Genomic Insights: QTL Mapping and GWAS for Stress Tolerance Genes

Genomics serves as the foundational blueprint for elucidating genetic variation underpinning crop tolerance to drought and heat stresses, enabling the identification of key loci through quantitative trait locus (QTL) mapping in biparental populations and genome-wide association studies (GWAS) in diverse germplasm panels [93]. These methodologies have systematically uncovered genomic regions governing yield stability and physiological traits under abiotic constraints across major crops, facilitating targeted breeding for enhanced resilience.

In wheat, QTL analyses have delineated multiple loci influencing grain yield under combined drought and heat, such as a prominent QTL on chromosome 4B that bolsters photosynthetic efficiency and root architecture [94]. Introgression of drought-tolerant QTLs from wild emmer relatives has validated their efficacy in elite cultivars [95], while GWAS incorporating phenomics traits like normalized difference vegetation index (NDVI) and canopy temperature has pinpointed markers on chromosomes 2A and 3B associated with cooler canopies, aligning with established drought-responsive genes [96].

Rice genomics highlights *qDTY* QTLs, including *qDTY12.1* on chromosome 12, which exert substantial effects on yield under upland drought and have been deployed via marker-assisted selection [97]. Recent GWAS leveraging UAV-derived dynamic drought responses has revealed novel loci modulating senescence kinetics [39]. For heat tolerance, genes like *TT1* (*thermotolerance1*) maintain pollen viability at elevated temperatures.

Maize studies, including large-scale GWAS, emphasize loci on chromosomes 7 and 8 enriched in dehydration-responsive element-binding (DREB) transcription factors and heat shock proteins in tolerant lines [98]. QTLs for anthesis-silking interval co-localize with abscisic acid (ABA) biosynthesis genes, mitigating reproductive delays under stress.

Sorghum, a resilient C4 crop, features cloned stay-green QTLs (*Stg1–Stg4*), with *Stg3* encoding an NAC transcription factor that delays senescence [99]. GWAS on diverse panels using drone phenotyping identified over 200 loci linked to biomass traits under drought.

In legumes like soybean and chickpea, GWAS has associated markers with canopy wilting and root traits, implicating stomatal regulation and ABA signaling genes [100]. Cotton heat tolerance QTLs near small heat shock proteins (HSPs) and transcription factors underscore fiber yield stability [101].

Collectively, these genomic investigations recurrently implicate core pathways: ABA signaling components (e.g., PYR/PYL receptors, SnRK2 kinases), HSP chaperones, transcription factor families (DREB, NF-Y, WRKY, NAC), and antioxidant enzymes [102,103]. Functional validation, such as overexpressing ABA receptors within QTL intervals, confirms enhanced drought survival [104].

Advancements extend to genomic selection (GS), which utilizes genome-wide markers to predict breeding values for polygenic traits. Integrating phenomics data into GS models, as in wheat yield forecasting under stress, improves predictive accuracy by capturing intermediate phenotypes [105]. Thus, genomics yields candidate genes and markers, bridging observable traits to molecular mechanisms, though functional elucidation necessitates complementary omics layers.

### 3.2. Transcriptomic Responses: Gene Expression Networks Under Stress

Transcriptomics, through RNA sequencing (RNA-Seq) or microarray profiling of mRNA expression, elucidates how plants reprogram gene expression in response to drought and heat stresses, highlighting differences between tolerant and susceptible genotypes. Extensive transcriptome studies across crops have illuminated regulatory networks and key genes activated during these stresses.

Under drought, tolerant genotypes typically exhibit rapid and robust induction of genes encoding osmoprotectants, late embryogenesis abundant (LEA) proteins, dehydrins, detoxification enzymes, and abscisic acid (ABA) signaling components compared to susceptible ones. For instance, in drought-tolerant wheat, ABA-responsive genes (e.g., *RAB18, RD29A*) and protective enzymes (e.g., glutathione S-transferases) are upregulated more swiftly [106,107]. In maize roots, tolerant lines uniquely induce genes for root elongation and aquaporins to sustain water uptake.

Heat stress induces widespread upregulation of heat shock proteins (HSPs) and their regulators, heat shock factors (HSFs). Tolerant rice varieties show elevated baseline and induced expression of small HSPs, chaperonins, and membrane-stabilizing genes, such as lipid desaturases. Transcriptomes of heat-stressed cotton anthers reveal sustained metabolic genes (e.g., sugar and energy pathways) correlating with pollen viability in resilient cultivars [108,109]. Heat also engages hormonal crosstalk, upregulating ABA-responsive transcription factors (e.g., ABFs/AREBs) and genes related to ethylene or brassinosteroids.

Combined drought–heat stress elicits unique expression profiles absent in individual stresses [15]. In barley flag leaves, this synergy induces ~1000 novel genes involved in protein repair and ubiquitination, emphasizing proteostasis, while amplifying HSF/HSP expression and suppressing photosynthesis-related genes more severely [110].

Key regulators include transcription factor families: DREB/CBFs for dehydration-inducible genes, bZIPs (e.g., ABFs) linking ABA to effectors, NACs and WRKYs for stress and senescence modulation, and HSFs for heat responses. Time-course RNA-Seq shows early activation (1–3 h) of regulators, followed by effectors like LEAs or HSPs [111,112]. A sunflower WRKY factor, upregulated only under combined stress, suggests mediation of drought–heat crosstalk [113].

Differential expression between genotypes reveals tolerance mechanisms: tolerant rice sustains *HSFA2* and HSPs during heat for enhanced protein and membrane stability [114,115]; drought-tolerant chickpea maintains proline (P5CS) and ABA (NCED) biosynthesis genes for osmoprotectant accumulation. High-resolution analyses uncover tissue specificity, with maize roots favoring ABA/cytokinin pathways for growth and leaves prioritizing HSF/HSPs and antioxidants.

Transcriptomic data enable co-expression networks identifying hubs, such as a soybean NAC factor enhancing drought tolerance upon overexpression [116]. They also refine QTL candidates: a rice drought-yield QTL gene (Dehydration Responsive Factor X) showed differential induction, validated transgenically.

In summary, transcriptomics delineates dynamic responses, confirming transcriptional regulation of ABA–SnRK2 and HSF–HSP pathways (e.g., *DREB2A* by ABA; *HSFA1/HSFA2* by heat) [117,118], while revealing crosstalk (e.g., *RD29A* in combined stress) [119] and ROS-scavenging genes in tolerant lines. This yields candidates for protein and metabolite-level scrutiny.

### 3.3. Proteomics: Stress Proteins and Post-Translational Adjustments

Proteomic profiling quantifies the proteome, the functional effectors of stress responses, offering insights into post-transcriptional and post-translational regulation where protein abundance diverges from transcript levels. Under drought and heat stress, proteomic analyses delineate differential protein accumulation in tolerant versus sensitive genotypes.

A hallmark of drought tolerance is the upregulation of late embryogenesis abundant (LEA) proteins and dehydrins, which function as molecular chaperones or hydrophilins to stabilize cellular structures during desiccation. For instance, comparative proteomics of drought-stressed barley revealed elevated dehydrin isoforms in leaves and crowns of tolerant cultivars, uncorrelated with mRNA induction, implicating translational or stability controls [120].

Heat stress elicits robust induction of heat shock proteins (HSPs), corroborating transcriptomic data. In heat-exposed wheat and rice flag leaves, tolerant lines exhibit pronounced accumulation of small HSPs (e.g., HSP17, HSP23), HSP70, and HSP90, mitigating protein aggregation compared to sensitive counterparts [121]. Proteomics further unveils post-translational modifications (PTMs), such as phosphorylation or oligomerization of HSPs, enhancing chaperone efficacy in tolerant genotypes.

Oxidative stress from drought and heat prompts elevated antioxidant and detoxification enzymes, including superoxide dismutase (SOD), ascorbate peroxidase (APX), glutathione peroxidase, catalase, and glutathione-ascorbate cycle components, constitutively or inducibly higher in tolerant plants [122]. In tomato under combined heat–drought, a tolerant accession sustained Cu/Zn-SOD and chloroplastic APX, correlating with reduced reactive oxygen species (ROS) [123]. Repair enzymes, such as DNA glycosylases and protein disulfide isomerases, also increase to ameliorate oxidative damage.

Metabolic reprogramming is evident: enzymes for osmolyte synthesis (e.g., betaine aldehyde dehydrogenase for glycine betaine, Δ1-pyrroline-5-carboxylate synthetase [P5CS] for proline) are upregulated, while energy-demanding pathways (e.g., Calvin cycle, nitrogen assimilation) are downregulated in sensitive genotypes. Tolerant rice maintains Rubisco activate and oxygen-evolving complex proteins under heat, prolonging photosynthesis [121,124].

Signaling and regulatory proteins, often invariant at transcript level, display PTM differences; abscisic acid (ABA) signaling kinases (SnRK2, CDPKs) and phosphatases exhibit enhanced phosphorylation in tolerant maize under drought, bolstering ABA transduction. Drought also induces alternative splicing, yielding distinct isoforms in tolerant lines [125].

For combined stresses, proteomics identifies multifunctional proteins: HSP101 accumulates exclusively in tolerant wheat, interfacing heat refolding with dehydration protection; thioredoxins and cyclophilins, elevated in tolerant chickpea, facilitate protein folding amid dual stressors.

The ABA-SnRK2 pathway manifests at protein level: PYR/PYL receptors accumulate or diversify in drought-tolerant canola. Phosphoproteomics demonstrates rapid PTM dynamics, e.g., heat-induced dephosphorylation of photosynthetic proteins and phosphorylation of chaperones/transcription factors in rice; LEA phosphorylation modulates interactions; ubiquitination escalates for proteasome-mediated clearance of misfolded proteins, being more efficient in tolerant Arabidopsis [126].

Proteomic discoveries inform breeding: a drought-associated LEA in peanut suggests enhancement targets; transgenic overexpression of LEAs/dehydrins confers drought resilience; a novel heat-induced enzyme in tolerant sorghum stabilizes thylakoids [127].

In summary, proteomics validates transcriptomic predictions, elucidates protein-centric regulations, and underscores chaperone/antioxidant accumulation as tolerance signatures, intertwined with ROS homeostasis. This transitions to metabolomics, examining downstream small-molecule effectors.

### 3.4. Metabolomics: Metabolic Reprogramming Under Drought and Heat

Metabolomics profiles small molecules that act as osmolytes, antioxidants, and signaling intermediates—the final effectors of stress tolerance. Under drought, tolerant genotypes accumulate compatible solutes such as proline, glycine betaine, trehalose, mannitol, and soluble sugars (glucose, fructose, sucrose) to maintain osmotic balance and stabilize macromolecules. For example, tolerant tomato accessions accumulate proline and raffinose to sustain turgor, while chickpea lines enrich sugars and sorbitol in stems [128]. Heat stress similarly promotes solute accumulation and lipid remodeling that preserve membrane integrity; tolerant wheat maintains polyunsaturated fatty acids and sterols, while grapevine increases flavanols and anthocyanins that scavenge reactive oxygen species (ROS) [129]. Under combined drought–heat, maize elevates γ-aminobutyric acid (GABA) and amino derivatives to buffer carbon and nitrogen metabolism, while sunflower boosts sucrose and malate for respiratory support. Recurrent tolerance-associated metabolites include proline, polyols (mannitol/sorbitol), glycine betaine, and raffinose, all repeatedly linked to resilience [130]. Adjustments in energy metabolism (malate, fumarate, citrate in the tricarboxylic acid, TCA cycle) and hormonal metabolites (abscisic acid, ABA; ethylene precursor 1-aminocyclopropane-1-carboxylic acid, ACC; cytokinins sustaining root growth) further differentiate tolerant from sensitive backgrounds [131,132]. Polyamines (putrescine, spermidine) and non-enzymatic antioxidants (ascorbate, glutathione, tocopherols, flavonoids) complement this defense, as observed in cotton and cucumber under combined stress [133,134].

To convert metabolite profiles into field-ready indicators, targeted and untargeted LC/GC–MS analyses should be paired with plot-level imaging—canopy temperature depression (CTD = T_air_ − T_canopy_), Photochemical Reflectance Index (PRI), and red-edge indices—across stress time courses. Hyperspectral features in the near-infrared (NIR), short-wave infrared (SWIR), and red-edge regions can act as spectral proxies for key metabolites when calibrated with parsimonious models (partial least-squares regression, regularized ensembles) and externally validated across sites and seasons. A pragmatic workflow is to (i) use satellite or unmanned aerial vehicle (UAV) series to flag stress windows; (ii) sample a subset of genotypes for leaf metabolomics at standardized times of day; (iii) fit and validate spectral–metabolite models; (iv) track yield components to quantify decision value [135]. When metabolite patterns consistently co-vary with yield stability, they constitute selection biomarkers. Mapping metabolite quantitative trait loci (mQTLs) and integrating with GWAS/TWAS links these profiles to genetic regulators, enabling metabolite-aware indices to be embedded into G × E × P pipelines for predictive modeling of drought–heat resilience. A comparative summary of key metabolite classes, functions, spectral proxies, and representative crops is provided in Table 3.

### 3.5. Key Pathways: ABA–SnRK2, HSF–HSP, and ROS Crosstalk

Multi-omics convergence highlights interconnected pathways underpinning drought–heat tolerance.

- ABA–SnRK2 pathway: Drought sentinel; ABA-receptor (PYR/PYL) binding relieves *PP2C* inhibition, activating SnRK2 kinases to phosphorylate ABF transcription factors, inducing responsive genes. Omics corroborate genomic variants in receptors/SnRK2; transcript induction of biosynthesis/ABFs; proteomic phosphorylation/accumulation; metabolic ABA surge. Extends to heat: ABA-deficient mutants vulnerable via stomatal dysregulation; *HSFA6b* bridges ABA-heat. Agricultural exploitation: ABA agonists preemptively activate responses, efficacious in fields albeit cost-limited [136].
- HSF–HSP network: Heat master regulators; HSFA1 trimerizes post-HSP90 release, amplifying HSFA2/HSP cascade for protein refolding/membrane protection. Multi-omics: Elevated baseline/inducible HSF/HSP in tolerant crops; sHSPs safeguard organelles. Drought synergy: HSP70 osmotic induction; PTMs (phosphorylation/oligomerization) modulate. Intersects ABA/ethylene/calcium; calmodulin activates HSFs; TOR influences HSP translation. Transgenics overexpressing HSP/HSF confer thermotolerance, tempered by growth penalties [137].
- ROS management: Drought/heat disrupt electron transport, generating superoxide/H_2_O_2_, damaging biomolecules. Tolerance via enzymatic (SOD/catalase/peroxidases) and non-enzymatic (ascorbate/glutathione/flavonoids) scavenging. ROS dual role: Low levels signal defenses (H_2_O_2_ activates TFs/ABA); tolerant balance signaling/excess. Crosstalk: ABA employs ROS in stomatal closure; chloroplast singlet oxygen triggers nuclear acclimation; HSPs stabilize scavengers/induce antioxidants [138].
- Ancillary: SnRK1 integrates sugar/energy; Ca^2+^ spikes activate CDPKs for ROS/ABA targets; lipid peroxides signal; MAPKs cascade to HSFs; SnRK2 osmotic sans ABA; *HSFA6b* ABA-responsive [139,140].

Holistic network where ABA orchestrates dehydration tolerance, HSF–HSP cellular integrity, and ROS redox homeostasis, supported by metabolic/redox feedback. Crop enhancement: Select for ABA sensitivity/chaperone induction without yield trade-offs; physiological screens (canopy temperature/membrane stability) [139].

## 4. G × E × P Integration and “Pixels-to-Proteins” Framework

**Figure 6:**
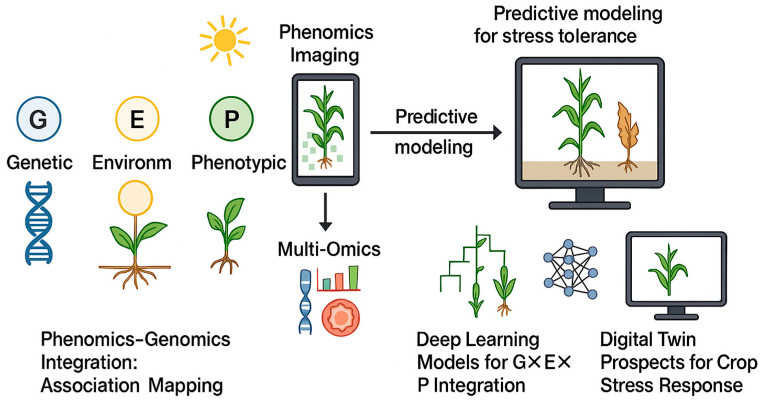
G × E × P integration via a pixels-to-proteins pipeline: harmonizing phenomics and multi-omics with Cal/Val and explainable AI to generate breeding and agronomy decisions.

Advancing plant breeding requires G × E × P pipelines that integrate genotype (G)—markers, QTLs, GWAS/TWAS hits—environment (E)—weather, soil, and management—and phenotype (P)—multi-scale imaging and plot-level traits—across time and scale. Figure 6 illustrates a pixels-to-proteins workflow in which canopy-level indices—canopy temperature depression, Photochemical Reflectance Index (PRI), red-edge and related spectral features—are co-collected with transcriptomic, proteomic, and metabolomic signatures. Standardized metadata and calibration/validation (Cal/Val) align sensor-derived signals with physiological ground truth. Integrated phenomic–omic features feed explainable machine learning models with uncertainty quantification to capture genotype × environment interactions. Model outputs inform three decision layers: breeding (selection indices; probability of superiority across target environments), agronomy (irrigation, nutrient, or shading prescriptions with confidence intervals), and research (hypothesis generation and active sampling). A digital-twin module simulates crop trajectories under alternative interventions and seasons and closes the loop through field validation and iterative model retraining.

### 4.1. Phenomics–Genomics Integration: Association Mapping

High-throughput field phenomics provides quantitative, heritable traits that power association analyses and expose loci governing drought–heat responses. In durum wheat, NDVI time-series derived from drone and ground imaging resolved stay-green QTL on chromosomes 2B/7A, co-localizing with chlorophyll-catabolism genes [141]. In rice, a UAV-derived drought-response index enabled GWAS that pinpointed recovery loci harboring ABA-signaling genes, linking canopy dynamics to regulatory variation [39].

Thermal and structural phenotyping further strengthens genetic dissection. In cotton and sorghum, drone thermography associated canopy temperature and biomass with genetic variation, delivering associations in sorghum [142]. For predictive breeding, incorporating UAV-based canopy temperature features increased genomic prediction accuracy for wheat yield under drought, demonstrating the value of phenomics priors in statistical models [143]. In parallel, envirotyping that uses remotely sensed weather and soil layers improved G × E modeling and genotype–environment matching across sites and seasons [144].

Conceptually, pixels-derived phenomics traits feed statistical genomics—phenomics-assisted genomics—to nominate markers and genes, followed by targeted validation and deployment. Consistent with this workflow, CRISPR/Cas9 editing of a UAV-GWAS candidate kinase in maize verified its causal role in drought tolerance, closing the loop from trait signal to gene function [145]. Best practice includes multi-environment replication with Cal/Val against physiological references (e.g., CTD, Fv/Fm, Ψ_leaf_), explicit reporting of stress windows, and uncertainty estimates to ensure transferability of associations into breeding decisions.

### 4.2. Multi-Omics + Phenomics Case Studies

Holistic G × E × P integration—combining multi-scale phenomics with genomics, transcriptomics, proteomics and metabolomics—illuminates mechanisms that underlie field performance. In a potato diversity panel, high-throughput stress phenotyping (imaging) was paired with transcriptomics and metabolomics, and correlation networks linked ABA-biosynthesis genes together with proline and raffinose accumulation to osmotic adjustment and delayed wilting under water deficit [146].

In wheat introgression lines, spectral traits and yield were integrated with GWAS and RNA-seq, revealing a drought-associated QTL that encodes a detoxification enzyme; this gene was overexpressed in tolerant lines and coincided with reduced ROS, providing a mechanistic bridge from locus to phenotype [147]. Proteome-level validation further tightens this link: GWAS candidates in the HSP family showed concordant abundance changes, and a kinase within a sorghum stay-green QTL exhibited stress-responsive phosphorylation in tolerant genotypes, consistent with enhanced chaperone signaling [148].

Model-based integration delivers actionable prediction. In maize, a hybrid model that combined CNN (convolutional neural network) features from UAV imagery with genomic markers improved genomic prediction of biomass under drought compared with either data stream alone [145]. A UAV–GWAS–transcriptome workflow that sampled canopy-temperature extremes mapped loci and recovered DEGs (differentially expressed genes), implicating aquaporins in sustaining transpiration and canopy cooling [149]. More generally, Bayesian networks, multi-layer machine learning models, and knowledge graphs provide scalable frameworks to assimilate heterogeneous data, linking field traits to metabolites, genes, and QTL while preserving causal hypotheses for downstream testing [150]. Across these case studies, best practice includes explicit Cal/Val against physiological references, standardized reporting of stress windows, and external validation to ensure that discoveries translate across sites and seasons.

### 4.3. Deep Learning Models for G × E × P Integration

Deep learning (DL) provides a flexible framework to integrate phenomics, genomics, and environmental variables by modeling complex non-linear interactions. In image–genomic prediction, convolutional neural networks (CNNs) extract canopy and architectural features from phenomics imagery (P), while dense layers encode genomic markers (G), and combined outputs predict yield or resilience. Environmental signals are implicitly captured within imagery, thereby embedding the G × E component without the need for explicit covariates. These architectures already improve drought-yield predictions by coupling spectral indices with marker-based models.

Beyond image-based pipelines, DL enables omics–phenomics bridging and multi-modal learning. Neural networks have been trained to predict drought-responsive transcript levels directly from hyperspectral reflectance, linking canopy spectra with molecular regulation [151]. Similarly, transcriptome-derived features at the seedling stage can be classified by neural nets to distinguish tolerant versus sensitive genotypes, supporting earlier and less invasive selection. More advanced frameworks aggregate inputs across domains—weather, soil, images, and genomic profiles—for trait or stress projections. For example, time-series UAV imagery combined with daily environmental records improves yield forecasts, while fusing soil-moisture signals with canopy images enhances drought detection.

A persistent challenge is the interpretability of deep networks. Emerging solutions include saliency maps, which highlight influential image regions, and attention mechanisms, which weight genomic loci or environmental variables. These techniques not only increase user trust but also uncover novel interactions—such as specific spectral band × weather variable combinations that forecast stress before visible symptoms, linked to membrane stability under vapor pressure deficit (VPD). Such explainable DL approaches help translate black-box models into biologically meaningful hypotheses, strengthening their role in breeding, agronomy, and decision-support systems.

### 4.4. Digital Twin Prospects for Crop Stress Response

Digital twins provide a virtual representation of crops or fields that update in near real time through the assimilation of imaging, sensor data, and simulations [152,153]. In principle, they embody G × E × P integration in silico, enabling stress forecasting and scenario testing. At the field scale, digital twins ingest satellite and UAV imagery (P) together with IoT environmental sensors (E) and genotype-parameterized crop models to predict yield and stress outcomes [154]. At the plant scale, twins can incorporate omics-derived physiological modules, such as ABA-mediated stomatal conductance regulation or HSP-driven canopy temperature control, linked to crop growth processes (water and carbon allocation) and gene networks underlying stress-induced senescence [155].

Although implementation remains nascent, prototypes illustrate the concept. Greenhouse-based digital twins have coupled imaging data with control systems for dynamic irrigation and climate regulation [156]. In open-field contexts, established crop models such as APSIM or DSSAT are being extended with real-time remote sensing inputs and genotype-specific coefficients, providing a steppingstone toward full digital twins. Such systems can simulate stress trajectories, explore irrigation scheduling, or assess the impact of heatwave onset on yield stability. They also support breeding ideotype design, for instance by evaluating how enhanced root hydraulics or cooler canopy traits would perform across environments.

Future development requires broader integration of remote sensing and weather records for establishing reliable baselines, together with extensions to predict extreme events. Ultimately, digital twins complement the progression from phenomics–genomic synergies for locus discovery, to multi-omics for trait dissection, to deep learning for data fusion, by adding a simulation layer that closes the loop between prediction and intervention. Early exemplars—such as UAV-led gene identification or omics-informed phenotypic modeling—underscore the potential of digital twins to foster proactive breeding and management strategies. This trajectory lays the foundation for truly data-enabled climate-smart agriculture (Table 4).

## 5. Translational Applications for Climate-Smart Agriculture

**Figure 7:**
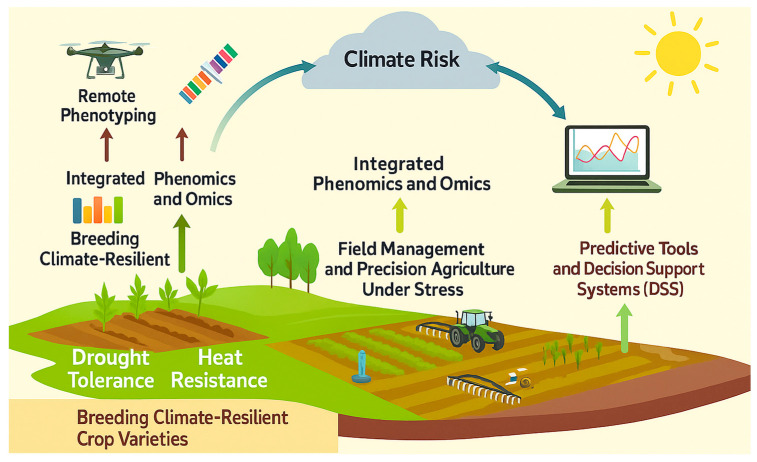
Phenomics–omics translation into breeding, field management, and decision-support systems (DSS).

Advances in remote-sensing phenomics and multi-omics are moving beyond discovery to enable climate-smart agriculture (CSA). These integrated approaches support three main applications: (a) crop breeding for drought–heat tolerance, where trait-linked genomic and metabolomic signatures accelerate selection; (b) field management and precision agronomy, where canopy indices (NDVI, CTD, PRI) inform irrigation, fertilization, and shading; (c) predictive modeling and decision-support systems (DSS) that combine phenomics, omics, environmental, and forecast data to guide proactive interventions. Such tools deliver tangible benefits including more resilient varieties, optimized resource use, and early stress warnings. Building on the methodological framework in Figure 6, which integrates phenomics, omics, and explainable AI, Figure 7 illustrates how these pipelines translate into actionable breeding programs, precision agronomy, and farm-level DSS.

Figure 7 shows the translation pathway from trait discovery to field applications. In practice, DSS integrate satellite and UAV indices with environmental and genetic layers to generate site-specific prescriptions, enabling farmers to act on stress forecasts and reduce yield losses. Effective deployment, however, requires calibrated thresholds, interoperability standards, and user-friendly delivery (e.g., mobile or SMS-based platforms for smallholders).

### 5.1. Breeding Climate-Resilient Crop Varieties

Breeding for drought and heat resilient cereals has long been impeded by the low heritability of yield under stress and the polygenic nature of the underlying physiology. The convergence of high-throughput phenotyping, genomics and accelerated generation advance is now delivering measurable genetic gain. Remote and proximal sensing of canopy temperature and normalized difference vegetation index (NDVI) have been fully embedded in the International Maize and Wheat Improvement Center (CIMMYT) wheat pipelines; selecting lines that maintain cooler canopies and higher NDVI in managed stress environments yields significantly greater stability than selection on grain yield alone [157,158].

Speed-breeding protocols that extend photoperiod in controlled environments allow five to six generations per year. When coupled with automated imaging, breeders can impose precise drought treatments on early generations, quantify shoot and root traits, and recycle only superior progeny, thus shortening the selection cycle by 30–40% [159]. Markers derived from phenomics genome-wide association studies (GWAS) and quantitative trait loci (QTL) mapping are now standard in rice and maize. Introgression of *qDTY* loci into Asian mega-varieties confers a 0.5–1.0 t ha^−1^ advantage in on-farm drought trials [160]. Genomic selection models trained with grain yield plus physiological covariates (e.g., canopy temperature, hyperspectral indices) increase predictive ability for stress-year yield by up to 30% and permit off-site selection, further compressing the breeding cycle [161].

High-resolution phenotyping also accelerates pre-breeding. In sorghum, imaging and genomics disentangled wild alleles for early-season chilling tolerance from linkage drag, enabling their introgression into tropical backgrounds [162]. Precision phenomics confirmed the positive effect of wild emmer segments on water-limited grain filling in elite wheat without a yield penalty [163]. The same platforms are uncovering novel adaptive traits. Thermal imaging combined with gas-exchange assays revealed genotypic variation in night-time stomatal conductance; lines that close stomata overnight conserve soil moisture and achieve superior daytime cooling. Hyperspectral estimation of stem carbohydrate dynamics identified genotypes able to remobilize stem reserves during terminal stress, sustaining kernel weight [164].

Multi-omics has pinpointed central regulators now being manipulated by transgenics and gene editing. Overexpression of *DREB1A* or HSP101 enhances drought or heat tolerance but may incur yield costs in benign seasons. CRISPR knockout of a *PP2C* negative regulator of ABA signaling in rice hastens stomatal closure and improves survival under acute drought [165]. Multiplex editing of heat-shock factor networks or promoter engineering for stress-inducible expression is feasible, as exemplified by ongoing edits of *GhHB17* in cotton to safeguard reproductive tissues during heat waves [166,167].

Because drought seldom occurs in isolation, breeding programs increasingly test under combined water and thermal stress at “climate-analog” sites. In pearl millet and maize, selection in high-temperature drought nurseries has produced hybrids that outperform older checks across erratic Sahelian seasons. Similar gains are reported for heat-tolerant spring wheat in South Asia and rice on the Indo-Gangetic Plain [168].

Collectively, integrating phenomics with genomic tools is transforming climate adaptation breeding from empirical selection to data-driven precision, delivering cultivars that secure 20–30% yield advantages in the world’s most vulnerable production zones.

Commercially deployed examples enabled by phenomics and multi-omics. Multiple programs have translated high-throughput phenotyping and omics-informed discovery into released, farmer-adopted varieties. In rice, drought-tolerant lines originating from marker/QTL pipelines have been deployed across Eastern India and South Asia through national systems, with on-farm advantages over local checks under water-limited conditions [65,160,168]. In wheat, canopy-temperature depression, hyperspectral traits, and thermal imaging have been used as indirect selection criteria to assemble heat- and drought-tolerant germplasm that national programs subsequently released, improving stability under late-season heat while maintaining performance in favorable seasons [53,54,55,66,67]. In sorghum, stay-green donor introgressions and canopy-development ideotypes—identified and tracked with field phenotyping—have underpinned drought-resilient materials used by breeding programs in semi-arid environments [40,50,51]. In maize, industry–public pipelines combining managed-stress trials, genomic selection, and field-based high-throughput phenotyping have delivered drought-tolerant hybrids that maintain yield under stress without sacrificing performance under optimal management [145,161]. Together, these cases illustrate a repeatable path from sensor-derived traits and omics loci to released varieties adopted at scale.

### 5.2. Field Management and Precision Agriculture Under Stress

Remote-sensing phenomics is now integral to climate-smart agronomy, translating sensor data into dynamic prescriptions that curb drought- and heat-induced yield loss [169]. Thermal and multispectral imagery captured by drones, satellites or pivot-mounted sensors drives variable-rate irrigation (VRI): canopy-temperature or crop water stress anomalies are mapped within seconds, so sprinklers boost flow only in water-deficient zones. Infrared-guided VRI in maize has saved ≈20% irrigation water without compromising yield, and NDVI-based prescription maps have been validated across the south-eastern United States [170].

Frequent Sentinel-2 revisits (≈5 d) enable early detection of incipient stress at landscape scale. Declines in NDVI, the Normalized Difference Moisture Index or solar-induced chlorophyll fluorescence trigger advisories that prompt irrigation, misting or shading before irreversible damage, with machine learning frameworks now delivering field-scale alerts several days ahead of visual wilting [171].

Sensor-guided variable-rate application extends beyond water: spectral or thermal cues delineate zones requiring foliar micronutrients, anti-transpirants, or targeted pesticides, reducing inputs while sustaining physiological activity during hot, dry spells. In orchards and vineyards, high-resolution thermal maps inform selective pruning or shade-cloth deployment and automate mist-fan initiation, balancing transpiration with fruit quality. Remote-sensing metrics also underpin financial risk management. Index-based drought insurance already uses regional NDVI or rainfall anomalies to trigger payouts, enabling farmers to re-sow or purchase fodder after severe events; integration of satellite soil-moisture products refines the correlation between index and yield loss, cutting basis risk and improving actuarial performance [172].

Decision-support systems fuse these datasets with weather forecasts and crop models. When a forecast heatwave coincides with maize anthesis, the system may recommend pre-irrigation or foliar boron to safeguard pollen viability, actions verified against sensor-derived growth stage and stress intensity. At watershed scale, evapotranspiration maps guide reservoir releases, while drought hot spots identified via vegetation indices focus relief efforts. Technologies are also migrating directly into farmers’ hands: smartphone-mounted thermal cameras and off-the-shelf NDVI drones enable rapid diagnosis of blocked drip lines or compaction-induced stress, allowing same-day remediation.

The synergy between management and genetics is clear—stress-tolerant cultivars maintain higher NDVI and cooler canopies longer, giving managers a wider decision window; conversely, sensing platforms expose thresholds at which even resilient genotypes need intervention. Together, precision phenomics and improved germplasm promise markedly better water productivity and yield stability in a warming, water-scarce world [172].

### 5.3. Predictive Tools and Decision-Support Systems (DSS)

Climate-smart agriculture is shifting from reactive responses to proactive, data-driven management. Multi-year satellite observations, weather forecasts, and phenomics now feed yield and stress forecasting that can flag risks (e.g., heat during flowering) weeks in advance, enabling pre-emptive actions such as targeted irrigation, stress-mitigation advisories, or import planning. On-farm DSS apps increasingly integrate forecast weather (E), crop genetics (G), and in-field or drone sensors (P) to tailor recommendations—adjusting irrigation cadence, foliar nutrition, and timing of inputs around heat waves to reduce stress [173].

Because heat and drought predispose crops to pests and diseases (e.g., spider mites, aflatoxin), modern DSS link abiotic stress maps with biotic risk alerts, directing scouting or prophylactic treatment to high-risk zones. At policy scales, integrated indicators (meteorological drought, soil moisture, crop condition) now improve drought declarations and disaster response, decreasing both missed events and false alarms. For long-term climate adaptation, G × E × P modeling and climate-scenario simulations identify trait packages and varieties suited to future heat and water regimes; climate-analog testing accelerates validation by trialing today in places that mirror tomorrow’s climate [174].

Digital twins—already maturing in controlled environments—predict short-term stress from VPD and radiation, triggering pre-emptive irrigation or misting. Field-scale twins that pair hydrological and crop growth models can forecast soil-moisture drawdown and phenology, scheduling irrigation precisely when it has the greatest yield impact [154]. In resource-limited settings, community DSS deliver simple, timely advice via SMS or radio, while experts fuse satellite vegetation indices, weather outlooks, and crowdsourced observations to power drought early warning and cropping-strategy pivots (e.g., shifting planting dates or varieties) [174]. Evidence from farms and national systems shows that these tools save water, reduce heat damage, and buffer food systems by improving reserve planning. Ultimately, adoption hinges on usability and demonstrated benefit; as data pipelines and models improve, predictive DSS will become a central pillar of climate resilience—sustaining yields amid rising drought and heat variability.

Conclusions and actionable priorities. (1) Standardization and benchmarks: publish open Cal/Val protocols, multi-site reference datasets, and robust metrics penalizing domain-shift failure. (2) Scale-aware causal inference: combine plot-level trials with landscape replication; use causal graphs/negative controls to separate exposure from tolerance. (3) Compound-stress realism: synchronize thermal peaks with water deficits at reproduction; report stage-specific thresholds and uncertainty. (4) Explainable decision systems: co-develop interpretable ML and fail-safes, embedding prescription rules auditable by agronomists. For breeders: prioritize CTD×PRI stability during flowering; verify with GWAS/TWAS-backed candidates. For agronomists: implement two time-of-day UAV thermal flights on stress days and act on pre-defined CTD/PRI thresholds. For data scientists: use time-blocked external validation and physics-constrained features.

## 6. Knowledge Gaps and Future Directions

Despite substantial progress, three priority gaps continue to constrain the impact of phenomics–omics integration for multi-stress resilience.

(1) Standards and shared benchmarks. Heterogeneous metadata, trait vocabularies, and file formats still impede cross-study synthesis, while limited pairing of imaging and molecular measurements hampers reuse at scale. Wider adoption of community standards, explicit Cal/Val protocols, and AI-assisted harmonization are necessary, together with paired phenomics–omics acquisitions that are versioned and citable. The immediate deliverable is an open, MIAPPE-compliant benchmark—combining imaging and omics with persistent DOIs and versioned labels—to enable fair method comparisons and reproducibility across sites and years.
(2) Climate-realistic, multi-scale modeling with explainability. Field crops experience compound, non-additive stresses whose dynamics span organs, canopies, and fields; yet many pipelines remain single-stress and site-specific, and state-of-the-art models are often opaque. Future work should couple multi-site trials that impose realistic drought–heat regimes with models that link organ-level physiology to canopy signals and field performance, while embedding biophysical constraints and reporting transparent attributions. The concrete deliverable is a multi-site compound-stress dataset aligned across organ–canopy–field scales, plus openly evaluated models that pass time-blocked external validation and publish attention/SHAP attributions keyed to biological priors [152,175].
(3) The “E” in G × E × P must move beyond coarse labels to stage-aware thresholds that are portable across climates and actionable in farms. Trials should quantify timing, intensity, and co-variation in stresses, and distill them into rules that breeders and agronomists can operationalize through decision support. The practical deliverable is a set of stage-specific stress thresholds validated in ≥3 contrasting climates, together with farmer-facing DSS pilots that report usability metrics and return-on-investment under commercial management [175].
(4) Regulatory and policy barriers. Field phenotyping increasingly relies on UAS/multi-sensor imaging, yet operations and data governance (ownership, privacy, cross-site sharing) impede multi-location trials and interoperability [48,64,74,142]. Varietal release and seed systems remain slow; aligning DUS/performance testing with climate-resilience traits and strengthening delivery pathways are emphasized in adaptation guidance [1,5]. Environmental permits and community consent can constrain heating/irrigation manipulations, and digital decision-support requires uncertainty disclosure and auditability. Targeted actions—enabling compliant UAS use, interoperable data standards, and streamlined release for resilience traits—would accelerate equitable adoption [74].

Despite significant progress, several knowledge gaps and challenges remain at the intersection of phenomics, omics, and stress tolerance, which guide future research directions. Addressing these will be crucial to fully realize the potential of integrated approaches for multi-stress resilience (Table 5).

By addressing these gaps, we will be better poised to develop the next generation of crops and tools that can withstand the increasingly erratic climate. The vision is that perhaps in a decade, breeders routinely use integrated phenomics-genomic analyses to create multi-stress tolerant crops, farmers have AI-driven apps that tell them exactly when and how to protect their crops from an impending stress, and policymakers have robust early warning to organize support—all pieces working in concert for climate-smart agriculture.

## 7. Conclusions

Climate change-driven drought and heat stress pose unprecedented risks to global crop production. The convergence of multi-scale remote-sensing phenomics with genomics, transcriptomics, proteomics, and metabolomics establishes a transformative “pixels-to-proteins” framework. By linking canopy-level indicators such as canopy temperature depression and hyperspectral indices to regulatory pathways and allelic variations, this integration enables both mechanistic understanding and practical prediction of crop resilience. Tangible progress has already been made: breeders are identifying stress-adaptive variants, agronomists are applying sensor-informed decision-support systems (DSS), and data scientists are developing models that bridge phenotypes and multi-omics signatures.

Three research priorities now stand out. First, the development of data standards and cross-platform interoperability is essential to harmonize satellites, UAVs, ground sensors, and omics datasets, under FAIR principles of sharing and reuse. Second, the design of spatiotemporal field trials that simulate realistic multi-stress environments is required to validate biomarkers and ensure translation beyond controlled conditions. Third, the field deployment of digital twins and AI-driven DSS must progress from greenhouse proof-of-concept to robust, scalable systems, with interpretability, uncertainty quantification, and participatory farmer validation at the core.

Stakeholder-specific pathways further illustrate the value of this integration. For breeders, omics-guided phenomics accelerates gene discovery, supports marker-assisted selection, and enhances genomic prediction accuracy under stress environments. For agronomists, real-time canopy indices and DSS inform irrigation scheduling, nutrient optimization, and adaptive stress management at the field scale. For data scientists, the abundance of multi-modal datasets opens new opportunities for interpretable machine learning and digital-twin frameworks tailored to agriculture.

Despite these advances, caution is warranted. Digital twins and AI models remain largely confined to controlled or simulated contexts, and their effectiveness under heterogeneous field conditions is unproven. Achieving climate-smart agriculture will require coordinated international collaboration, robust data integration pipelines, and large-scale participatory validation in farmers’ fields. Only by uniting breeders, agronomists, and data scientists within an interoperable, field-ready ecosystem can the promise of phenomics–omics integration be realized to safeguard crop productivity and global food security in an increasingly variable climate.

## Figures and Tables

**Table 3:** Metabolite signatures and spectral proxies linked to drought–heat resilience.

Metabolite Class	Function In Stress Tolerance	Spectral Proxy (Indicative Features)	Representative Crops/Examples
Compatible solutes (proline, glycine betaine, trehalose, mannitol/sorbitol)	Osmotic adjustment; macromolecule stabilization; partial ROS buffering	NIR/SWIR water-sensitive bands (~1450–1510 nm, ~1940 nm); red-edge shifts; PRI context	Tomato, chickpea, maize; engineered glycine betaine in non-accumulators [128,129,130]
Antioxidants (ascorbate, glutathione, tocopherols; flavonols/anthocyanins)	ROS scavenging; membrane protection	UV–blue for flavonoids; red-edge position; PRI dynamics	Grapevine, cotton; tolerant lines sustain pools [129,135]
TCA/GABA shunts (malate, fumarate, citrate; GABA)	Respiratory priming; carbon/nitrogen buffering under combined stress	SWIR water/leaf chemistry features; time-series red-edge	Rice (TCA priming), maize (GABA surge) [131]
Polyamines (putrescine, spermidine)	Membrane stabilization; ROS mitigation; stress signaling	Indirect proxies via pigment/water-related bands; requires calibration/validation (Cal/Val)	Cucumber; exogenous application improves heat resilience [135]
Hormonal metabolites (ABA, ACC; cytokinins)	Stomatal closure (ABA); heat/ethylene signaling (ACC); root maintenance (cytokinins)	Indirect via CTD/PRI/red-edge combined with environmental covariates	ABA increase under drought; ACC under heat; tolerant lines maintain cytokinins [132]

**Table 4:** Case Studies of Phenomics-Multi-Omics Integration in Crop Breeding.

Crop	Integration Approach	Key Findings|Outcome	Outcome
Rice	UAV NDVI + Transcriptomics	Identified ABA loci for yield stability	Improved varieties
Maize	Satellite LST + Genomics	QTLs for G × E interactions	Enhanced prediction models
Wheat	Hyperspectral + Proteomics	HSP markers for heat tolerance	Breeding acceleration
Soybean	AI fusion (pixels-to-proteins)	Metabolite predictions	Precision management

**Table 5:** Knowledge Gaps and Future Directions in Phenomics–Multi-Omics Integration.

Gap	Description	Proposed Direction	Potential Impact
Data Standardization	Lack of uniform formats across platforms	Develop ontologies and AI harmonization	Improved cross-study comparisons
Combined Stress Models	Underrepresentation of multifactorial stresses	Multi-omics field trials with DL	Better real-world predictions
Accessibility in Developing Regions	High costs limit adoption	Low-cost sensors and open-source tools	Global equity in breeding
Digital Twins Implementation	Limited validation in diverse environments	Simulation-based breeding pipelines	Accelerated variety development

## Data Availability

Data are available through request to the corresponding author.

## Abbreviations

The following abbreviations are used in this manuscript:

ABA	Abscisic Acid
ACC	1-Aminocyclopropane-1-carboxylic Acid (ethylene precursor)
APX	Ascorbate Peroxidase
BRDF	Bidirectional Reflectance Distribution Function
Cal/Val	Calibration/Validation
CAT	Catalase
CDPK	Calcium-Dependent Protein Kinase
CT	Canopy Temperature
CTD	Canopy Temperature Depression
CWSI	Crop Water Stress Index
DSS	Decision-Support System
DT	Digital Twin
EVI	Enhanced Vegetation Index
Fv/Fm	Maximal Quantum Yield of PSII (Photosystem II)
g_s_	Stomatal Conductance
GABA	γ-Aminobutyric Acid
GSD	Ground Sampling Distance
GWAS	Genome-wide Association Study
G × E × P	Genotype × Environment × Phenotype
HSF	Heat Shock Factor
HSP	Heat Shock Protein
iWUE	Intrinsic Water-Use Efficiency
JA	Jasmonic Acid/Jasmonates
LAI	Leaf Area Index
LEA	Late Embryogenesis Abundant proteins
LiDAR	Light Detection and Ranging
LST	Land Surface Temperature
ML	Machine Learning
mQTL	Metabolite Quantitative Trait Loci
NDRE	Normalized Difference Red-Edge Index
NDVI	Normalized Difference Vegetation Index
NDWI	Normalized Difference Water Index
NIR	Near-Infrared
OEC	Oxygen-Evolving Complex
P5CS	Δ^1^-Pyrroline-5-Carboxylate Synthetase
PRI	Photochemical Reflectance Index
PTM	Post-Translational Modification
QTL	Quantitative Trait Locus/Loci
RGB	Red–Green–Blue imaging
ROS	Reactive Oxygen Species
Rubisco	Ribulose-1,5-bisphosphate Carboxylase/Oxygenase
SA	Salicylic Acid
SIF	Solar-Induced Fluorescence
SnRK2	Sucrose Non-Fermenting-1-Related Protein Kinase 2
SOD	Superoxide Dismutase
SWIR	Short-Wave Infrared
T_air_	Air Temperature
T_canopy_	Canopy Temperature
TCA	Tricarboxylic Acid cycle
TPE	Target Population of Environments
TWAS	Transcriptome-Wide Association Study
UAS	Unmanned Aircraft System
UAV	Unmanned Aerial Vehicle
UQ	Uncertainty Quantification
VI	Vegetation Index
VPD	Vapor Pressure Deficit
WUE	Water-Use Efficiency
Ψ_leaf_	Leaf Water Potential
